# Challenges of using evidence in managerial decision-making of the primary health care system

**DOI:** 10.1186/s12913-023-10409-7

**Published:** 2024-01-05

**Authors:** Marjan Hedayatipour, Sina Etemadi, Somayeh Noori Hekmat, Alisadat Moosavi

**Affiliations:** 1 Department of Healthcare Management, Policy and Economics, Faculty of Management and Medical Information Sciences, Kerman University of Medical Sciences, Kerman, Iran; 2 Department of Medical Library & Information Sciences, Faculty of Management and Medical Information Sciences, Kerman University of Medical Sciences, Kerman, Iran

**Keywords:** Decision-making, Evidence, Management, Primary healthcare

## Abstract

**Background:**

Managerial Evidence-Based Decision-Making [EBDM] in the primary is a systematic approach that directs the decision-maker in a conscientious, explicit, and judicious utilization of reliable and best evidence based on the professional experiences and preferences of stakeholders and patients from various sources. This study aimed to investigate the challenges primary healthcare managers encounter while undertaking decision-making processes.

**Method:**

A systematic review was conducted in 2022 with the aim of identifying and collecting all qualitative articles pertaining to evidence-based decision-making in the primary healthcare system. To achieve this, a meticulous search was conducted using the relevant keywords, including primary health care and evidence-based decision making, as well as their corresponding synonyms, across the databases Web of Science, Scopus, and Pubmed. Importantly, there were no limitations imposed on the timeframe for the search. To carefully analyze and consolidate the findings of this systematic review, the meta-synthesis approach was employed.

**Results:**

A total of 22 articles were assessed in this systematic review study. The results revealed the main categories including evidence nature, EBDM barriers, utilizing evidence, decision-makers ability, organizational structure, evidence-based, EBDM support, communication for EBDM, evidence sides, EBDM skill development, public health promotion, and health system performance improvement.

**Conclusion:**

The primary healthcare system is crucial in improving health outcomes and ensuring access to healthcare services for all individuals. This study explored the utilization of evidence-based EBDM within the primary healthcare system. We identified five key dimensions: causal, contextual, and intervening conditions, strategies, and consequences of EBDM as a core phenomenon. The findings will help policymakers and administrators comprehend the importance of evidence-based decision-making, ultimately leading to enhanced decision quality, community well-being, and efficiency within the healthcare system. EBDM entails considering the best reliable evidence, and incorporating community preferences while also exploiting the professional expertise and experiences of decision-makers. This systematic review has the potential to provide guidance for future reforms and enhance the quality of decision-making at the managerial level in primary healthcare.

**Supplementary Information:**

The online version contains supplementary material available at 10.1186/s12913-023-10409-7.

## Introduction

Healthcare organizations function within dynamic, competitive, and uncertain contexts that constantly transform, necessitating the need for adaptability in order to thrive and succeed in achieving their goals and mission [1]. In the realm of healthcare organizations, the processes of decision-making and management hold a paramount position due to their exceptional significance, thereby being capable of yielding consequential outcomes that possess the potential to greatly influence the direction and trajectory of these organizations [2]. If the managers of healthcare organizations apply the best evidence in their administration and decision-making processes, they have the potential to enhance the likelihood of the organization's triumph by executing efficacious choices [3, 4].

Evidence-based decision-making [EBDM], is a methodical and systematic approach that directs the decision-maker in a conscientious, explicit, and judicious utilization of reliable and best evidence taking into consideration the preferences of stakeholders and patients from various sources. This approach entails a meticulous manner to enhance the probability of achieving favorable outcomes in high-quality health service delivery and patient satisfaction. The process involves the precise identification of the issue and the problem, followed by a comprehensive search for evidence. Subsequently, the evidence is collected and subjected to critical evaluation. Once this evaluation is complete, the decision-maker proceeds to carefully select and apply the evidence in the decision-making process. Finally, the results of the decisions made are evaluated to assess their effectiveness [5–8].

Provided that decisions are made within healthcare organizations without taking into account the most reliable and best evidence, it could result in detrimental outcomes. These adverse consequences encompass a lack of effectiveness, efficiency, and justice. This, in turn, leads to a decrease in productivity and overall inefficiency within society, as well as an escalation in healthcare costs. Moreover, it diminishes the quality of health services and overall performance within organizations, while also posing risks for adverse side effects. Additionally, it fosters conflicts of interest among stakeholders within health organizations, forfeiting opportunities to enhance the health of individuals within the community and ultimately eroding public trust in the healthcare system [9].

Evidence denotes the collection of information, data, or facts that a manager can utilize in order to arrive at an optimal decision. Evidence encompasses summaries of analyzed data and information pertaining to a specific domain, various forms of research specifically reviews, the professional perspectives of experts in the respective field, and, lastly, taking into consideration the values and preferences of patients and the other stakeholders [10–15].

Primary care centers serve as the first line of contact between the general population and the healthcare system. The responsibility of the primary care system in order to prevent, maintain, and enhance public health is of significant importance. Furthermore, considering the changes in the healthcare landscape and society, as well as the public's health service needs and expectations, it is imperative that experts in this field possess current information and evidence and utilize them to the maximum extent when making decisions. With EBDM at the forefront of healthcare, the health system can take effective measures to achieve the health-related goals of all community members [16].

Evidence-based decision-making is a professional process that relies on the best and most trustworthy evidence while considering expertise and taking into account patients' preferences and values [5]. It is a recommended approach to decision-making that utilizes theories, experience, knowledge, and information to improve managers' performance and decision-making [17, 18]. By incorporating evidence into the management decisions of the primary healthcare system, we can anticipate improvements in public health and an increase in the quality of healthcare services. When managers utilize evidence in their decision-making, the outcomes become more reliable and of higher quality, allowing them to play a vital and influential role in guiding and leading different aspects of the primary healthcare system [16].

However, it is essential for healthcare managers to critically evaluate the evidence and ensure that it is based on sound scientific principles and valid data. Evidence-based decision-making concerning public health matters serves as a benchmark for best practices within the system, enabling managers to optimize patient care and health promotion, enhance the performance of healthcare organizations, and avoid resource wastage.

In recent years, there has been a growing body of research focused on the utilization of evidence within different facets of the healthcare system. Within this context, studies are about investigating the process of incorporating evidence into health system decision-making [3, 7, 9, 19, 20], evidence-based management practices, and the availability of evidence sources within hospitals [21, 22], cross-sectional studies have also explored the identification and generation of evidence, sources of access to evidence, and the capacity for utilizing and evaluating evidence within the primary healthcare system [23–30]. However, despite the production of evidence within the health system, particularly through research efforts, there are significant challenges in effectively knowledge translation, leveraging knowledge brokers, and utilizing evidence [12, 31–33].

Given the importance of managerial decisions and their impact on health outcomes, as well as the significance of the primary healthcare system, it is crucial for managers and decision-makers within this field to base their decisions on the best available evidence. Nevertheless, they face obstacles in doing so. Therefore, this study was undertaken to investigate the challenges associated with the process of utilizing evidence in the management decisions of the primary healthcare system. Employing the grounded theory framework, this study examines the causal, contextual, and interventional conditions, strategies, and consequences of this process, drawing insights from studies conducted globally.

## Method

Systematic reviews are meticulously designed and conducted exploration of the existing literature. Their purpose is to comprehensively search, identify, evaluate, and consolidate all the pertinent and reliable research findings. The goal of a systematic review is to comprehensively locate and synthesize research that bears on a particular question using organized translucid and repeatable procedures at each step in the process [34]. The purpose of this systematic review and meta-synthesis was to ascertain the challenges encountered when incorporating evidence into managerial decision-making in PHC and strategies for its promotion in 2022.

### Search

Three databases were searched with no limitation of date, PubMed, Scopus, and Web of Science databases. A manual search in the Google Scholar search engine was used to complete and retrieve articles that were missed. The complete search process endeavor was conducted by an expert in library and information sciences specialist (AM). The search terms included two main concepts, "Evidence-Based Decision-Making" and "Primary Health Care". These phrases were adapted for use in each database. The search strategy was designed through the SPIDER tool. Since this systematic review was conducted using a qualitative research design, SPIDER^1^ as an alternative search strategy tool for qualitative/mixed methods was used instead of the PICO Search Tool (suitable for quantitative studies) [35] (Table 1).

**Table 1:** SPIDER Search Strategy

SPIDER Tool	Search Terms
S [Sample]	Primary Health Care OR PHC
PI [Phenomenon of Interest]	[Evidence Based Administration[Title/Abstract]] OR [Evidence Informed Administration[Title/Abstract]] OR [Evidence Based Management[Title/Abstract]] OR [Evidence Informed Management[Title/Abstract]] OR [Evidence Informed Practice[Title/Abstract]] OR [Evidence Based Practice[Title/Abstract]] OR [Evidence Based Decision Making[Title/Abstract]] OR [Evidence Informed Decision Making[Title/Abstract]] OR [Evidence Informed Policy Making[Title/Abstract]] OR [Evidence Based Policy Making[Title/Abstract]]
D [Design)]	All qualitative designs
R [Research type]	Qualitative study

[S AND P Of I]

When formulating a search strategy, a search tool is used as the organizer framework to determine terms according to the primary concepts outlined in the search query, particularly in situations where it is unfeasible to include an experienced information specialist as a member of the review team. The PICO Tool places emphasis on the population, intervention, comparison, and outcomes of a (typically quantitative) article. It is commonly utilized to identify components of clinical evidence for systematic reviews in evidence-based medicine and is verified by the Cochrane Collaboration [36]. However, certain search terms such as "control group" and "intervention" lack relevance to qualitative research, which traditionally does not incorporate control groups or interventions, and as a result, may not adequately facilitate the finding of qualitative research [37]. The PICO Tool currently does not include terms related to qualitative research or specific qualitative designs. In practice, it is often modified to "PICOS" where the "S" represents the study design. This modification helps limit the number of irrelevant articles. In order to address this limitation, a new search tool called "SPIDER" was developed. The SPIDER Tool is designed specifically to identify relevant qualitative and mixed-method studies. The addition of the "design" and "research type" categories to the SPIDER Tool enhances its ability to identify qualitative articles while eliminating irrelevant PICO categories such as the "comparison" group [35]. Following the SPIDER parameters, the search strategy was as follows:

### Inclusion criteria

- Studies published in English
- Qualitative studies including [but not limited to], phenomenology, grounded theory, ethnography, case studies, and thematic analysis studies addressing primary healthcare managers’ experiences, Opinions, and perceptions of EBDM.
- Qualitative studies about the processes and nature of managerial evidence-based decision-making in the primary healthcare setting.

Overall, the review included studies that addressed the experiences, perceptions, challenges, consequences, and strategies to improve evidence-based decision-making of managers, policymakers, and primary healthcare researchers who had implemented and experienced the EBDM approach in any type of primary healthcare institution or organization in any environment at the global level.

### Data extraction

Using the formulated search strategy, all articles on evidence-based management decision-making were retrieved without a specific time frame. Abstracts identified were imported into the Endnote. The duplicate articles were selected and removed. MH (primary reviewer) and SE (secondary reviewer) independently reviewed the abstracts for each paper using the eligibility criteria described above. Upon initial screening, the majority of the articles were excluded for the following reasons; not in English, and not about the managerial decision-making process in primary healthcare (PHC). Afterward, full-text articles were retrieved, MH and se independently reviewed the studies again and the following criteria were used to further exclude papers such as studies that did not use a qualitative methodology, did not about processes of managerial decision-making, or didn't discuss specifically about primary healthcare setting. The excluded studies were archived, along with the reason for exclusion. To ensure the reliability of the data collection process, MH and SE had several face-to-face discussions to reach a consensus on studies. Any disagreement between the two data collectors was resolved by a third researcher (SN).

### Quality appraisal

The quality of the included studies was assessed by both reviewers independently using the critical appraisal skills program (CASP) checklist which assesses the risk of bias, and whether the study design, data collection, and analysis were appropriate for the study [38]. The assessment classified the quality of the studies at three strong, moderate, and weak levels. The eligible articles, i.e., strong and moderate articles, were included in the systematic review.

### Data analysis and meta-synthesis

Given the qualitative nature of the data extracted from the studies, the meta-synthesis approach was used to analyze and consolidate the results of the systematic review. Compilation of qualitative findings is crucial for the advancement and progression of knowledge. Hence, the meta-synthesis of qualitative studies performs as a method that fosters the development of knowledge by amalgamating qualitative discoveries and phenomena that are of significance to the discipline [39]. Meta-synthesis entails the interpretation, integration, and inference of the process evaluation components derived from all the identified studies. Following thorough discussion and consensus among the reviewers, hypotheses are generated based on these findings [40].

The data were synthesized with an inductive approach. Data analysis was conducted using the grounded theory structure [41, 42]. This approach is an initial exploration of the available research body in order to extract the full theoretical implication from a well-chosen set of published studies [43]. In the classic Grounded theory approach, the researcher starts the investigation with pure data with no available theoretical background or framework about the phenomena.

Grounded theory (GT) is a precise systematic inductive method to understanding the social process through analyzing qualitative data and permitting the analyst to propose important ideas and develop a substantive theory that is compatible and consistent with empirical observation. Therefore, GT has been adopted as a recommended methodology for qualitative studies' analysis, content comparison, and theory generation [44].

In the endeavor of formulating a substantive theory through the utilization of grounded theory (GT), Creswell (2012) proposes that the focus should be placed on the process rather than the consequences. In the realm of GT research, Strauss and Corbin (1998) [45]. Define a process as "a sequence of actions and interactions among people and events pertaining to a topic". Consequently, the analysis of managerial evidence-based decision-making in primary healthcare was approached as a social process, and it was conducted in accordance with three prescribed steps [41, 46]: 1. Open coding—the establishment of preliminary categories of information regarding the phenomenon; 2. Axial coding—the identification of a core category and the determination of its relationships with the other identified categories; 3. Selective coding—the development of a theory aimed at elucidating the aforementioned relationships.

The data obtained from pertinent existing textual instances underwent a process of encoding through the application of a constant comparative analysis technique. The codes that emerged from this analysis were connected to overarching concepts, which were further organized into sub-categories and categories. These categories were subsequently classified into broader categories, enabling an exploration of the various casual conditions, core phenomena, intervening conditions, contexts, consequences, and strategies.

Moreover, the data and results related to EBDM within the PHC from the articles were extracted using a researcher-made checklist that determined the title, purpose, authors, place of publication, year of publication, journal, methodology, and results of the EBDM-related studies. Meta-synthesis was performed on the data collected from 22 articles using MAXQDA 2020 Software. Subsequently, a description of the managerial EBDM process in PHC is also presented.

## Result

**Fig.1:**
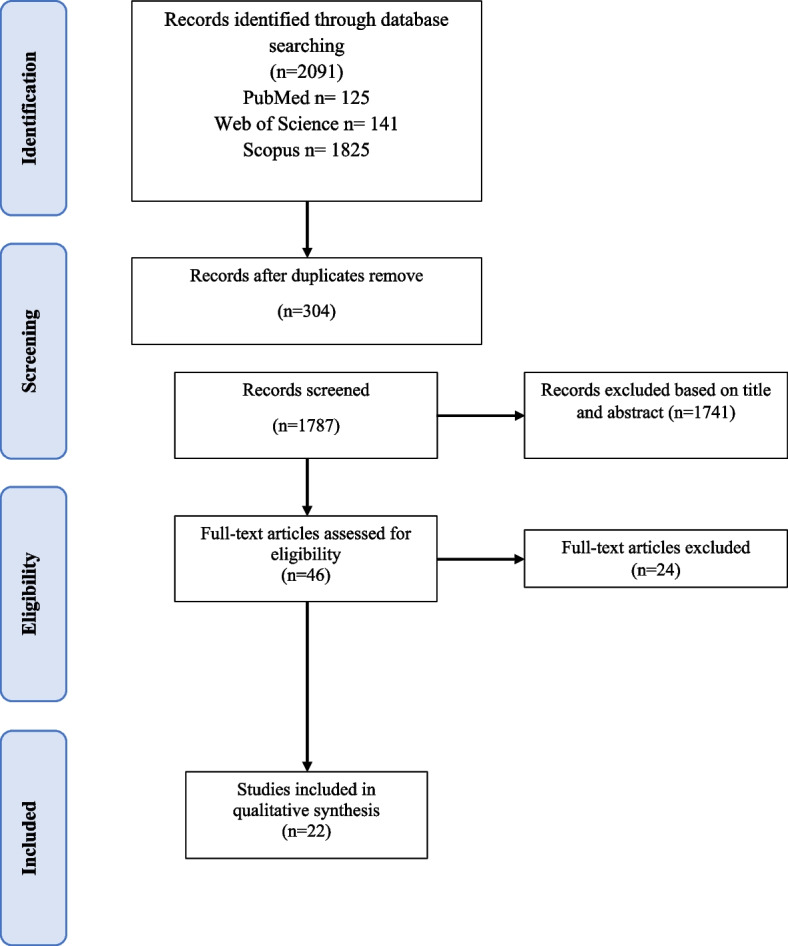
Prisma flow diagram

Accordingly, a total of 22 articles were selected for the final review (Fig. 1).

**Fig. 2:**
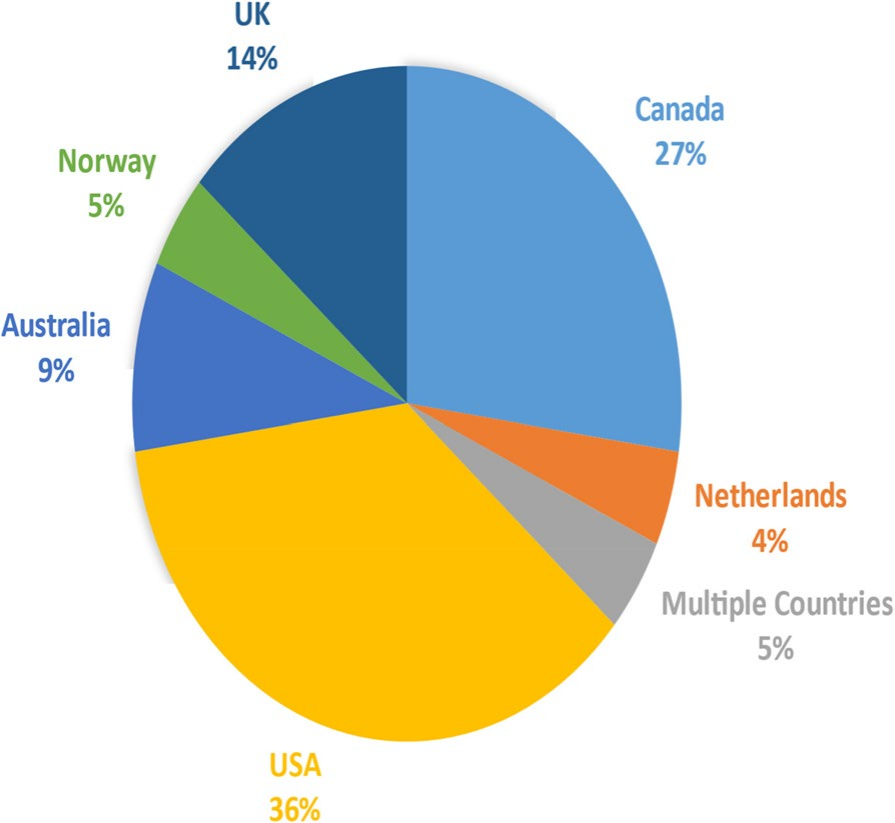
Frequency distribution of studies in different countries

Finally, 22 studies met the criteria for entering the review. Of all reviewed articles, 8 articles are in the United States [36 percent], 6 articles in Canada [27 percent], 2 articles in Australia [9 percent], 1 article from multiple countries including “Germany, Austria and Switzerland” [5 percent], 3 articles from the UK [14 percent], 1 article from Norway [5 percent] and 1 article from the Netherlands [5 percent] has been done (Fig. 2).

**Fig. 3:**
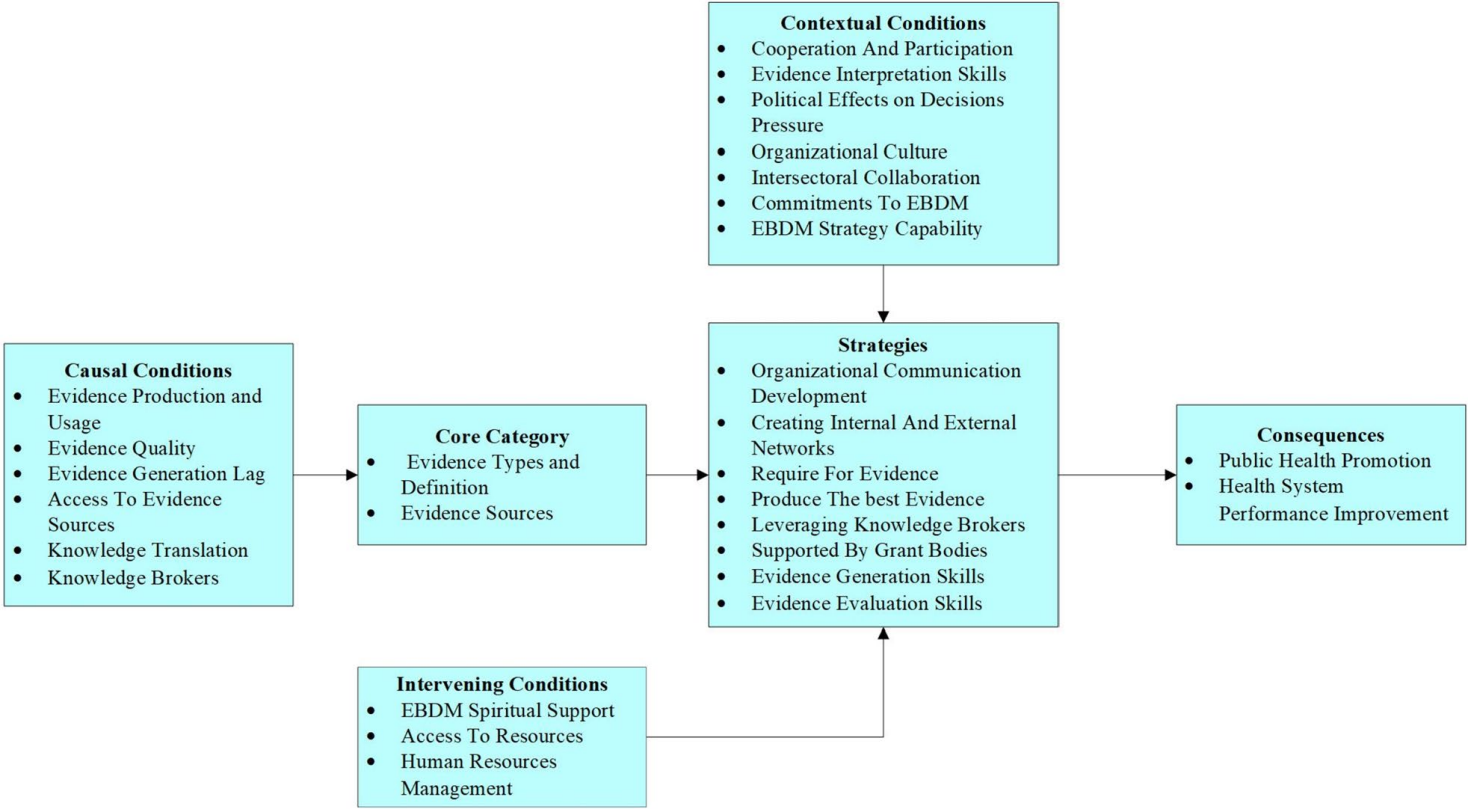
EBDM process Grounded Theory framework

Ultimately, 22 studies were reviewed. Codes, categories, and sub-categories were determined according to the statements extracted from the studies and related to the objectives (Table 2). Furthermore, the synthesis of results within the framework of the grounded theory model is illustrated (Fig. 3).

**Table 2:** Codes extracted in the systematic review and meta-synthesis of studies

Main Classifications	Main Categories	Sub-Categories	Code
**Causal Conditions**	Evidence Nature	Evidence Production and Usage	Evidence Producers [47–52]
			Evidence Users [48, 49, 53–59]
		Evidence Quality	Evidence Providers Skills [35, 48, 49, 51, 53–55, 58, 60]
			Evidence Importance [50, 53, 54, 56, 59, 61–63]
	EBDM Barriers	Evidence Generation Lag	Sufficient Time to Produce Evidence [57, 58, 60, 64]
			Sufficient Time to Use Evidence [49, 54, 58, 61, 62, 65]
		Access to Evidence Sources	Facilitating Access Importance [47, 51, 52, 54, 56, 59, 60]
			Providing Electronic Access [35, 51, 54, 56, 57, 59, 61, 66]
	Utilizing evidence	Knowledge Translation	Knowledge Translation Importance [47, 53, 56, 62, 64, 67]
			Knowledge Translation Process [35, 47, 54, 59–61, 63]
		Knowledge Brokers	Knowledge Broker's Importance [47, 58, 61]
			Type of Knowledge Brokers [62, 64]
**Contextual Conditions**	Decision-Makers’ Ability	Cooperation and Participation	Cooperation and Participation Importance [35, 51, 53, 55, 64, 66]
			Types of Cooperation And Partnership [50, 51, 53, 57–59, 65]
		Evidence Interpretation Skills	Comprehension And Analysis Skills [58, 67]
			Type of Interpretation [60]
	Organizational Structure	Political Effects on Decisions Pressure	Variable Impact [56, 58]
			Type of Impact [52, 54]
		Organizational Culture	Organizational Culture Importance [35, 47, 49, 52–54, 57, 63, 66, 67]
			Create an Organizational Culture [47, 53, 57, 59, 61, 66]
		Intersectoral Collaborations	Intersectoral Collaborations Importance [52, 56, 58, 67]
			Intersectoral Collaborations Circumstance [35, 62, 66]
		Commitments to EBDM	Commitment’s Importance [56, 65]
			Type of Commitments [53]
		EBDM Strategy Capability	Promote EBDM Planning [49, 55]
			EBDM Planning Framework [49, 51]
**Core Category**	Evidence-based	Evidence Types and Definition [54, 63]	
		Evidence Sources	Sources Types [50, 60]
			Sources Competency [53, 60, 62]
**Intervening Conditions**	EBDM Support	EBDM Spiritual Support [47, 61, 65, 66]	
		Access to Resources	Financial and Time Resources Importance [49, 55, 58, 61, 64]
			Resource Generation [47, 52, 59, 65–67]
		Human Resources Management	Workforce Development [52, 56, 59, 66]
			Provide/Use of Evidence Motivation [54, 55, 61, 62, 65]
**Strategies**	Communication for EBDM	Organizational Communication Development [47, 51–53, 55–59, 62, 67, 68]	
		Creating Internal and External Networks [47, 49, 53, 55, 59, 62, 64]	
	Evidence Sides	Require for Evidence [49, 56, 58, 59]	
		Produce The Best Evidence [47, 50, 55, 58, 63]	
		Leveraging Knowledge Brokers [56, 59, 64]	
		Supported by Grant Bodies [47, 57, 64]	
	EBDM Skill Development	Evidence Generation Skills	Training for Generation [48, 49, 51–55, 57, 59, 65, 66]
			Evidence Summary [53, 60, 63, 66]
		Evidence Evaluation Skills	Evidence Evaluating Importance [49, 53, 55, 61]
			Evidence Evaluation Capacity Building [48, 49, 54, 61]
**Consequences**	Public Health Promotion [47, 49, 51, 53, 55, 57, 64, 68]		
	Health System Performance Improvement [47, 49, 51, 53, 55, 57, 64, 68]

### Evidence nature

In general, out of the 22 final articles included in the study, some articles have directly addressed the evidence producers [47–52], the evidence users [48, 49, 53–59], the Evidence Providers Skills [35, 48, 49, 51, 53–55, 58, 60], and Evidence Importance [50, 53, 54, 56, 59, 61–63]. The evidence was examined from the perspective of both evidence producers and evidence users. The significant concept pertains to the request made by decision-makers in order to facilitate the process of EBDM via the demand for evidence. Therefore, it is imperative that researchers or knowledge brokers produce credible and beneficial evidence, enabling the making of high-quality decisions. Moreover, It has also been posited in the reviewed literature that the evidence could be obtained from various types of research methodologies (such as quantitative, qualitative, mixed methods, and specifically reviews, meta-synthesis, and meta-analysis) that could potentially be utilized by administrators and decision-makers in the primary healthcare setting.

### EBDM barriers

Some reviewed articles also addressed the barriers that EBDM confront. These challenges included Sufficient Time to Produce Evidence [57, 58, 60, 64], Sufficient Time to Use Evidence [49, 54, 58, 61, 62, 65], Facilitating Access Importance [47, 51, 52, 54, 56, 59, 60], and Providing Electronic Access [35, 51, 54, 56, 57, 59, 61, 66]. The issue under consideration is the existence of adequate time for producing, evaluating, and using evidence. Generally, the time required to conduct studies to produce evidence is relatively longer than the time available to decision-makers. The other imperative issue is access to evidence sources, especially electronic resources, or facilitating access to these resources. Facilitating the accessibility of diverse databases and developing appropriate tools to generate evidence, this act of facilitation transpires.

### Utilizing evidence

Some articles addressed the knowledge translation importance [47, 53, 56, 62, 64, 67], and the Knowledge Translation Process [35, 47, 54, 59–61, 63]. They stated that one of the ways to build EBDM capacity is the existence of an effective knowledge translation mechanism in the planning, decision-making, and functioning of public health organizations. Knowledge translation and transfer processes are significantly important to meet the expectations of decision-makers to use research evidence in public health decision-making and overcome individual, organizational, and contextual barriers to supporting, advancing, and sustaining EBDM. The concepts of knowledge broker's importance [47, 58, 61], and type of knowledge brokers [62, 64] were discussed the importance of knowledge transfer, and how to implement it. The results indicated that the presence of knowledge brokers at work to complete a rapid review of evidence is a particular process. Knowledge mediation is a reliable process of translating organizational knowledge to support EBDM. The transfer and dissemination of knowledge through knowledge brokers facilitate knowledge transfer, promote decision-makers skills, and improve performance.

### Decision-makers ability

Some articles addressed the cooperation and participation of EBDM in public health. This concept includes cooperation and participation importance [35, 51, 53, 55, 64, 66], and types of cooperation and partnership [50, 51, 53, 57–59, 65]. In these studies, the interpretation of engagement and collaboration, notwithstanding the involvement of stakeholders, particularly patients and community members in the process of arriving at decisions, also signifies cooperation and participation on the side of the decision-maker as well as the researchers and knowledge brokers, with the aim of generating and utilizing evidence. It is imperative that such collaboration be undertaken to enhance the quality of decision-making. A few articles highlighted the comprehension and analysis skills of the decision-makers [58, 67] and Type of evidence interpretation [60]. They discussed the dimensions of evidence understanding and analysis skills. If health organization managers lack statistical data analysis skills or interpret the evidence in a way that contradicts its nature, the EBDM process will turn into a challenging task.

### Organizational structure

Some articles addressed the influence of political issues on the decision-making process [56, 58] and discussed the variability and type of these effects on political issues [52, 54]. The decision-making process is often affected by health policies, and in some cases, decision-makers have limited representation and authority in making decisions. However, some studies indicate the organizational culture importance [35, 47, 49, 52–54, 57, 63, 66, 67], and create an organizational culture [47, 53, 57, 59, 61, 66]. These articles highlighted the support of a talented, visionary, and strongly motivated senior health official as a requirement for EBDM to achieve its goals, activities, and success. Moreover, some other important concepts on this subject were intersectoral collaborations importance [52, 56, 58, 67], intersectoral collaborations circumstance [35, 62, 66], commitments importance [56, 65], type of commitments [53], promote EBDM Planning [49, 55], and EBDM planning framework [49, 51]. In an effort to effectively implement the EBDM process in the public health setting, the studies highlighted the require for interdisciplinary cooperation not only between health researchers but also between health researchers from several fields and community doctors and professionals from different fields. Without the dedication and commitment to employ this procedure and organize the framework for strategizing and planning, we cannot anticipate triumph and advancement in this particular approach.

### Evidence-based

Articles reviewed indicated evidence types and definitions [54, 63], source types [50, 60], and source competency [53, 60, 62] pointed out that EBDM is a process in which multiple sources of information are consulted before making a decision regarding the provision of services. In this process, research evidence is integrated into the decision-making process to inform and guide public health policy and program planning. Studies emphasized the importance of receiving systematic reviews, executive summaries of research, and clear statements of implications for practice from health service researchers in order to facilitate the integration of research evidence into decision-making.

### EBDM support

Four articles investigated the importance of support and how to create spiritual support EBDM process [47, 61, 65, 66]. The provision of Spiritual Support, encouragement, and motivation to evidence producers and employees by leaders and decision-makers of the organization can serve as a significant catalyst in the process of evidence production and utilization. Additionally, some articles addressed financial and time resources importance [49, 55, 58, 61, 64], and Resource Generation [47, 52, 59, 65–67]. They showed that one of the factors affecting evidence-based performance in the public health domain is having different budget sources. Furthermore, Workforce Development [52, 56, 59, 66], and Provide/Use of Evidence Motivation [54, 55, 61, 62, 65] were considered effective dimensions. However, it is important to note that these dimensions can also be seen as shortcomings if they encounter difficulties, but can be employed as a means for enhancement to improve under different circumstances.

### Communication and networking for EBDM

Some articles discussed organizational communication development [47, 51–53, 55–59, 62, 67, 68] and creating internal and external networks [47, 49, 53, 55, 59, 62, 64]. One of the facilitating factors of EBDM in the primary healthcare system is networking to communicate and share information and evidence. To solicit evidence from researchers or, conversely, to furnish decision-makers with the evidence generated, it is advisable to enhance interpersonal and organizational communication, as well as communication with external entities, by implementing pragmatic methodologies and networking.

### Evidence sides

The results of the reviewed studies demonstrated that the parameters of utilizing evidence encompass Require For Evidence [49, 56, 58, 59], Produce The best Evidence [47, 50, 55, 58, 63], Leveraging Knowledge Brokers [56, 59, 64], and Supported By Grant Bodies [47, 57, 64]. strategies aimed at enhancing and promoting the utilization of evidence in managerial decision-making comprise establishing a mechanism and fostering a culture that emphasizes the importance of decision-makers appealing evidence. Moreover, this evidence ought to be generated in response to the demands of policymakers and managers, and should be presented in a manner that is comprehensible, highly pertinent, up-to-date, reliable, and applicable. Simultaneously, the involvement of knowledge brokers plays a crucial role in revitalizing and enhancing this process. Conversely, by considering valuable organizational credits and The growth of grant bodies for the EBDM, organizations are likely to exhibit greater adherence to this process.

### EBDM skill development

Some studies acknowledged that EBDM skill development includes training for generation [48, 49, 51–55, 57, 59, 65, 66], evidence summary [53, 60, 63, 66], evidence evaluating importance [49, 53, 55, 61], and evidence evaluation capacity building [48, 49, 54, 61]. In general, these articles highlighted education and training as one of the requirements, prerequisites, and facilitators of EBDM. likewise creating evidence summaries is one of the main strategies to facilitate decision-making and effective use of evidence. Whereas policymakers and managers utilize evidence, it is imperative to adopt a critical viewpoint and thoroughly assess the evidence in order to effectively ascertain and amalgamate the best evidence, subsequently enabling them to make an informed decision based on it.

### Public health promotion

The results showed that the use of reliable evidence in decision-making will promote individual and public health Public Health Promotion [47, 49, 51, 53, 55, 57, 64, 68]. The cause for this occurrence may be attributed to the advancements made in the realm of primary healthcare decision-making. Consequently, the implemented health programs, as well as the overall policies and services provided in this sector, have experienced enhancements.

### Health system performance improvement

The articles discussed the health system performance improvement [47, 49, 51, 53, 55, 57, 64, 68] as a general consequence. The review of the articles showed that one of the main outcomes of using EBDM in the primary healthcare system is to improve performance, effectiveness, efficiency, and quality of health service delivery.

## Discussion

EBDM is an approach to decision-making that relies on the most reliable, up-to-date, and best evidence. In the course of this approach, managers and decision-makers acquire and assess data and information from various sources, including scientific research, expert perspectives and opinions, and empirical data, as well as the preferences of stakeholders. EBDM endeavors to guarantee that decisions are grounded in factual and best information, as well as objective evaluations, rather than being influenced by subjective biases or personal convictions. This evidence-based approach enables decision-makers to make informed choices based on the best evidence, thus elevating the overall quality and effectiveness of decision-making processes.

To this end, this study aimed to identify the challenges of using evidence in the primary healthcare system as well as the ways to promote the use of evidence in managerial decision-making in this area. By reviewing 22 related studies, challenges, infrastructures, contextual conditions, strategies, and potential consequences were identified. The findings revealed the causal, contextual, and intervening conditions, and strategies needed for the establishment and promotion of EBDM, and identified the possible consequences.

Flexibility in producing evidence is very important because it makes it possible to face emerging issues [47]. Managers of health organizations can obtain evidence from various sources, including research, examination of the type of problem and its causal conditions, hospital information, and data, ethical and behavioral issues, management skills and experiences, and values and preferences of patients and beneficiaries, socio-political development programs, unique organizational and environmental characteristics, and analysis of the organization’s internal and external environment [47–52, 54–58, 67]. Most of the studies highlighted scientific and reliable research as the best source of evidence [50, 53, 54, 56, 59, 61–63]. On the other hand, the language of the evidence should be such that the decision-makers can use it more easily and effectively [60]. Some studies showed the potential sources of evidence include organizational resources, managers’ experiences, research products, facts, information, environmental and external data, stakeholders, and social factors [69–74]. academic evidence, conferences, internal organizational feedback, internal and external standards, and organizational rules and regulations [74]. A group of studies also indicated that the internet, access to databases, organizational websites, and online library systems lead to the development of organizational infrastructure and improve access to evidence [75–77].

Making research findings more widely available to primary healthcare decision-makers is likely to be beneficial. Policymakers reported that summaries and systematic reviews were often difficult to access. In other words, the studies conducted are often not presented efficiently to inform the issues related to policies, programs, and strategies [78–80]. Creating a culture of EBDM, critical thinking in solving issues and problems, searching for evidence, and promoting creative behavior in the organization have a positive and significant effect on performance. This means that the multiplicity of ways to support, innovate, motivate, and encourage employees will promote evidence-based practices. The results of other studies were consistent with the findings of the present study [81–84].

More than half of the articles assessed in this systematic review highlighted the importance of organizational culture and its causal conditions [47, 49, 52–54, 57, 59, 61, 63, 66, 67]. Leaders of healthcare organizations can serve as endogenous catalysts for creating and promoting an EBDM culture. Besides, long-term and consistent engagement of senior leaders with staff in the effective use of evidence can enhance the prominence and durability of EBDM. Leaders must believe that good decisions must be based on evidence. Strong leadership can facilitate an organizational culture that is more supportive of change and more willing to challenge deep-rooted attitudes [85–87].

The data from the reviewed studies indicated that one of the methods and tools for promoting EBDM is continuous knowledge translation and transfer in the field of primary healthcare. Knowledge translation contributes to making evidence available to public health professionals and organizations as well as all levels of government to advance national public health priorities [47, 53, 54, 56, 60–63, 66, 68]. Knowledge translation is an effective strategy for strengthening the acceptance and application of research results. Knowledge translation is defined as the production, exchange, synthesis, and ethical application of knowledge in the complex system of interactions between researchers and users to accelerate the acquisition of benefits from research. Knowledge translation can also contribute to improving community health, promoting health services and outcomes, and strengthening the healthcare system [88–93].

Knowledge transfer as one of the effective components of the evidence-use process facilitates meeting the expectations of the use of research evidence in public health decisions. The availability of tools and the role of knowledge brokers to support the EBDM process are known to be very important as confirmed in other studies conducted in this field [47, 58, 61, 62, 64]. According to these studies, knowledge transfer is a conscious action related to actively transferring knowledge and creating insights, assessments, experiences, or skills through verbal or non-verbal tools that improve decision-making [94–99].

Creating opportunities for interaction between managers and researchers is key to promoting the use of research evidence in policy-making and decision-making. Policymakers often seek advice from researchers, but sometimes cannot find the expertise they need and tend to resort to people in their contact list [100, 101]. On the other hand, researchers found that the participation of policymakers in their research projects is valuable, but they were often unsure of how to identify the right people. Moreover, many studies have recommended the creation of integrated evidence generation and consultation teams in the form of R&D centers in institutions operating in the primary healthcare system. Thus, with the cooperation and coordination of the members of the centers, up-to-date, reliable, and effective evidence is produced in the required and different areas and made available to the decision-makers at a suitable time or even before the occurrence of crises to prevent their consequences [47, 53, 55, 64]. According to some studies, the public health workforce lacks adequate research skills and critical evaluation skills, and more formal and advanced training on EBDM concepts, tools, technologies, and applications is needed [49, 102, 103].

Evidence-based public health, described as the integration of science-based interventions with community preferences to improve population health, has been widely expanded using community protection guidelines. Identifying evidence-based practices in public health contributes to creating an underlying and operational environment that supports and facilitates evidence-based public health [104]. Formulating policies and making effective health and evidence-based decisions; responding to public health emergencies; selecting, implementing, and evaluating cost-effective interventions; and the allocation of human and financial resources in health organizations, despite the agreement that decisions should be rational and based on data and evidence, also lead to the improvement of health outcomes [67]. The use of evidence in decisions and performance leads to the improvement of the quality of decisions and time and cost management. The effectiveness of EBDM can be improved by promoting it in public health departments and health sector decision-makers. Researchers also receive effective results and feedback to produce evidence [64, 79]. This study was conducted with some shortcomings, including the unavailability of the full text of all the articles in the systematic review, the multiplicity of databases in the countries, the differences in different health systems in various countries, and the inaccessibility of some databases in Iran. However, by synthesizing the data extracted from studies on EBDM in the primary healthcare system, the present study presented significant evidence to improve the EBDM process.

## Conclusion

The primary healthcare system is essential as it serves as individuals' first point of contact, providing comprehensive and accessible care, and promoting early intervention, disease prevention, and community health promotion. It plays a crucial role in improving health outcomes and ensuring equitable access to healthcare services for all individuals. High-quality decision-making in this area holds significant importance. This study investigation scrutinized the utilization of Evidence-Based Decision-Making [EBDM] by administrators within the primary healthcare system on a global scale. This analysis encompassed an evaluation of five key aspects pertaining to the core phenomenon of evidence-based decision-making. These five dimensions encompass causal, contextual, intervening, strategies, and consequences; utilization of these dimensions offers us an all-encompassing perspective on EBDM. The analysis of the studies included in this systematic review will help policymakers, administrators, and decision-makers in the realm of primary healthcare to Understand the nature and significance of using evidence in their decision-making process. This will enable them to employ the best information and efficacious approaches to leverage data and attain desirable outcomes, which would in turn enhance the quality of decision-making, foster community well-being, and optimize the efficacy of the healthcare system. EBDM is a systematic approach that entails utilizing the best available evidence when making determinations in the realm of public health. This approach leads to community involvement and takes into account community preferences, while also exploiting the professional expertise and experiences of decision-makers. Overall, the perception generated through this research has the potential to enhance the quality of the decision-making process within the primary healthcare system and serve as a roadmap for future reforms and promotion.

### Supplementary Information

**Additional file 1: Table A1.** SPIDER Search Strategy.

**Additional file 2: Table A2.** The bibliographic report of the studies included in the systematic review.

## Data Availability

The data in the study is comprised of previous research articles. A full list of articles is available from the corresponding author.

## Abbreviations

EBDM: Evidence-Based Decision-Making
PHC: Primary Healthcare
GT: Grounded theory
PRISMA: Preferred Reporting Items for Systematic Reviews and Meta-Analyses
PICO: Patient, Intervention, Comparison, Outcome
SPIDER: Sample, Phenomenon of Interest, Design, Evaluation, and Research Type

## Footnotes

1 Sample, Phenomenon of Interest, Design, Evaluation, and Research Type.
