# Supplementary material for: Challenges of using evidence in managerial decision-making of the primary health care system
Source: BMC Health Serv Res. 2024 Jan 5;24:38. doi: 10.1186/s12913-023-10409-7 (PMC10770934; doi:10.1186/s12913-023-10409-7)
Supplement: Supplementary file 1 — Additional file 1: Table A1. SPIDER Search Strategy. [file 12913_2023_10409_MOESM1_ESM.docx]

**Title: “Challenges of using evidence in managerial decision-making of the primary health care system”**

**Table A1 SPIDER Search Strategy**

| SPIDER Tool | Search Terms |
| --- | --- |
| S [Sample] | Primary Health Care OR PHC |
| PI [Phenomenon Of Interest] | [Evidence Based Administration[Title/Abstract]] OR [Evidence Informed Administration[Title/Abstract]] OR [Evidence Based Management[Title/Abstract]] OR [Evidence Informed Management[Title/Abstract]] OR [Evidence Informed Practice[Title/Abstract]] OR [Evidence Based Practice[Title/Abstract]] OR [Evidence Based Decision Making[Title/Abstract]] OR [Evidence Informed Decision Making[Title/Abstract]] OR [Evidence Informed Policy Making[Title/Abstract]] OR [Evidence Based Policy Making[Title/Abstract]] |
| D [Design)] | All qualitative designs |
| R [Research Type] | Qualitative Study |

[S AND P Of I].

**PUBMED**

((((((((((evidence Based Administration[Title/Abstract]) OR (evidence informed Administration[Title/Abstract])) OR (evidence Based management[Title/Abstract])) OR (evidence informed management[Title/Abstract])) OR (evidence informed Practice[Title/Abstract])) OR (evidence Based Practice[Title/Abstract])) OR (evidence Based decision making[Title/Abstract])) OR (evidence informed decision making[Title/Abstract])) OR (evidence informed Policy making[Title/Abstract])) OR (evidence Based Policy making[Title/Abstract])) AND ((PHC[Title/Abstract]) OR (primary health care[Title/Abstract]))

N= 125

## SCOPUS

## ( TITLE-ABS-KEY ( "evidence Based Administration" ) OR TITLE-ABS-KEY ( "evidence informed Administration" ) OR TITLE-ABS-KEY ( "evidence Based management" ) OR TITLE-ABS-KEY ( "evidence Based management" ) OR TITLE-ABS-KEY ( "evidence informed Practice" ) OR TITLE-ABS-KEY ( "evidence Based Practice" ) OR TITLE-ABS-KEY ( "evidence Based decision making" ) OR TITLE-ABS-KEY ( "evidence informed decision making" ) OR TITLE-ABS-KEY ( "evidence Based Policy making" ) OR TITLE-ABS-KEY ( "evidence informed Policy making" ) AND TITLE-ABS-KEY ( phc ) OR TITLE-ABS-KEY ( "primary health care" ) )

N=1825

**WOS**

1- "evidence Based Administration" (Topic) or "evidence informed Administration" (Topic) or "evidence Based management" (All Fields) or "evidence Based management" (Topic) or "evidence informed Practice" (Topic) or "evidence Based Practice" (Topic) or "evidence Based decision making" (Topic) or "evidence informed decision making" (Topic) or "evidence Based Policy making" (Topic) or "evidence informed Policy making" (Topic)

2- "Primary health care" (Topic) or PHC (Topic)

3- #1 AND #2

N=141
