# Supplementary material for: Challenges of using evidence in managerial decision-making of the primary health care system
Source: BMC Health Serv Res. 2024 Jan 5;24:38. doi: 10.1186/s12913-023-10409-7 (PMC10770934; doi:10.1186/s12913-023-10409-7)
Supplement: Supplementary file 2 — Additional file 2: Table A2. The bibliographic report of the studies included in the systematic review. [file 12913_2023_10409_MOESM2_ESM.docx]

**Title: “Challenges of Using Evidence in managerial decision-making of the Primary Health Care System”**

**Table A2 The bibliographic report of the studies included in the systematic review**

| \| publication \| Country \| Title \| author \| number \| \| --- \| --- \| --- \| --- \| --- \| \| 2020 \| Canada \| Public Health Network: Canada’s National Collaborating Centres: Facilitating evidence-informed decision-making in public health \| Alejandra Dubois (1) \|  \| \| 2020 \| Netherlands \| Four normative perspectives on public health policy-making and their preferences for bodies of evidence \| Casper Schoemaker (2) \|  \| \| 2018 \| Germany, Austria and Switzerland \| Facilitating evidence uptake: development and user testing of a systematic review summary format to inform public health decision-making in German-speaking countries \| Laura K. Busert (3) \|  \| \| 2018 \| Washington \| Building capacity for evidence-based public health: reconciling the pulls of practice and the push of research \| Ross C.Brownson (4) \|  \| \| 2015 \| USA \| Implementing administrative evidence based practices: lessons from the field in six local health departments across the United States \| Kathleen Duggan (5) \|  \| \| 2015 \| Colorado \| Peer Reviewed: Promoting Evidence-Based Decision Making in a Local Health Department, Pueblo City–County, Colorado \| Anna, Hardy (6) \|  \| \| 2015 \| Canada \| Challenges of partnership research: insights from a collaborative partnership in evidence-informed public health decision making \| Robyn Traynor (7) \|  \| \| 2014 \| Australia \| Understanding evidence: a statewide survey to explore evidence-informed public health decision-making in a local government setting \| Rebecca Armstrong (8) \|  \| \| 2014 \| Canada \| Tools to support evidence-informed public health decision making \| Jennifer Yost (9) \|  \| \| 2013 \| Australia \| Knowledge translation strategies to improve the use of evidence in public health decision making in local government: intervention design and implementation plan \| Rebecca Armstrong (10) \|  \| \| 2013 \| Norway \| From knowledge to action in public health management: experiences from a Norwegian context \| Lille fjell (11) \|  \| \| 2013 \| NewYork \| Factors affecting evidence-based decision making in local health departments \| Collette Sosnowy (12) \|  \| \| 2012 \| Scotland \| Experiences of knowledge brokering for evidence-informed public health policy and practice: three years of the Scottish Collaboration for Public Health Research and Policy \| Helen Frost (13) \|  \| \| 2012 \| USA \| Peer reviewed: tools for implementing an evidence-based approach in public health practice \| Julie Jacobs (14) \|  \| \| 2012 \| Canada \| Building capacity for evidence informed decision making in public health: a case study of organizational change \| Leslea Peirson (15) \|  \| \| 2012 \| Scotland and England \| Capturing complex realities: understanding efforts to achieve evidence-based policy and practice in public health \| Katherine Smith (16) \|  \| \| 2011 \| Canada \| Evidence-informed decision making in a public health setting \| Megan Ward (17) \|  \| \| 2010 \| Ireland \| Translating evidence into practice: a shared priority in public health? \| Helen McAneney (18) \|  \| \| 2009 \| Washington \| Evidence-based public health: a fundamental concept for public health practice \| Ross C.Brownson(19) \|  \| \| 2007 \| Canada \| Public Health Decision-Makers' Informational Needs and Preferences for Receiving Research Evidence \| Maureen Dobbins (20) \|  \| \| 2005 \| USA \| Evidence-based public health policy and practice: promises and limits \| Laurie Anderson (21) \|  \| \| 2003 \| USA \| Strengthening capacity in developing countries for evidence-based public health: the data for decision-making project \| Marguerite Pappaioanou (22) \|  \| |
| --- | --- | --- | --- | --- | --- | --- | --- | --- | --- | --- | --- | --- | --- | --- | --- | --- | --- | --- | --- | --- | --- | --- | --- | --- | --- | --- | --- | --- | --- | --- | --- | --- | --- | --- | --- | --- | --- | --- | --- | --- | --- | --- | --- | --- | --- | --- | --- | --- | --- | --- | --- | --- | --- | --- | --- | --- | --- | --- | --- | --- | --- | --- | --- | --- | --- | --- | --- | --- | --- | --- | --- | --- | --- | --- | --- | --- | --- | --- | --- | --- | --- | --- | --- | --- | --- | --- | --- | --- | --- | --- | --- | --- | --- | --- | --- | --- | --- | --- | --- | --- | --- | --- | --- | --- | --- | --- | --- | --- | --- | --- | --- | --- | --- | --- | --- |

**REFERENCES:**

1. Dubois A, Lévesque M. Public Health Network: Canada’s National Collaborating Centres: Facilitating evidence-informed decision-making in public health. Canada Communicable Disease Report. 2020;46(2-3):31.

2. Schoemaker CG, van Loon J, Achterberg PW, den Hertog FRJ, Hilderink H, Melse J, et al. Four normative perspectives on public health policy-making and their preferences for bodies of evidence. Health Res Policy Syst. 2020;18(1):94.

3. Busert LK, Mütsch M, Kien C, Flatz A, Griebler U, Wildner M, et al. Facilitating evidence uptake: development and user testing of a systematic review summary format to inform public health decision-making in German-speaking countries. Health Res Policy Syst. 2018;16(1):59.

4. Brownson RC, Fielding JE, Green LW. Building Capacity for Evidence-Based Public Health: Reconciling the Pulls of Practice and the Push of Research. Annual review of public health. 2018;39:27-53.

5. Duggan K, Aisaka K, Tabak RG, Smith C, Erwin P, Brownson RC. Implementing administrative evidence based practices: lessons from the field in six local health departments across the United States. BMC Health Serv Res. 2015;15:221.

6. Hardy AK, Nevin-Woods C, Proud S, Brownson RC. Promoting Evidence-Based Decision Making in a Local Health Department, Pueblo City-County, Colorado. Prev Chronic Dis. 2015;12:E100.

7. Traynor R, Dobbins M, DeCorby K. Challenges of partnership research: Insights from a collaborative partnership in evidence-informed public health decision making. Evid Policy. 2015;11(1):99-109.

8. Armstrong R, Waters E, Moore L, Dobbins M, Pettman T, Burns C, et al. Understanding evidence: a statewide survey to explore evidence-informed public health decision-making in a local government setting. Implement Sci. 2014;9:188.

9. Yost J, Dobbins M, Traynor R, DeCorby K, Workentine S, Greco L. Tools to support evidence-informed public health decision making. BMC Public Health. 2014;14:728.

10. Armstrong R, Waters E, Dobbins M, Anderson L, Moore L, Petticrew M, et al. Knowledge translation strategies to improve the use of evidence in public health decision making in local government: intervention design and implementation plan. Implement Sci. 2013;8:121.

11. Lillefjell M, Knudtsen MS, Wist G, Ihlebæk C. From knowledge to action in public health management: experiences from a Norwegian context. Scandinavian journal of public health. 2013;41(8):771-7.

12. Sosnowy CD, Weiss LJ, Maylahn CM, Pirani SJ, Katagiri NJ. Factors affecting evidence-based decision making in local health departments. Am J Prev Med. 2013;45(6):763-8.

13. Frost H, Geddes R, Haw S, Jackson CA, Jepson R, Mooney JD, et al. Experiences of knowledge brokering for evidence-informed public health policy and practice: Three years of the Scottish Collaboration for Public Health Research and Policy. Evid Policy. 2012;8(3):347-59.

14. Jacobs JA, Jones E, Gabella BA, Spring B, Brownson RC. Tools for implementing an evidence-based approach in public health practice. Prev Chronic Dis. 2012;9:E116.

15. Peirson L, Ciliska D, Dobbins M, Mowat D. Building capacity for evidence informed decision making in public health: a case study of organizational change. BMC Public Health. 2012;12:137.

16. Smith KE, Joyce KE. Capturing complex realities: Understanding efforts to achieve evidence-based policy and practice in public health. Evid Policy. 2012;8(1):57-78.

17. Ward M. Evidence-informed decision making in a public health setting. Healthc Manage Forum. 2011;24(1 Suppl):S8-16.

18. McAneney H, McCann JF, Prior L, Wilde J, Kee F. Translating evidence into practice: A shared priority in public health? Soc Sci Med. 2010;70(10):1492-500.

19. Brownson RC, Fielding JE, Maylahn CM. Evidence-based public health: a fundamental concept for public health practice. Annual review of public health. 2009;30:175-201.

20. Dobbins M, Jack S, Thomas H, Kothari A. Public health decision-makers' informational needs and preferences for receiving research evidence. Worldviews Evid Based Nurs. 2007;4(3):156-63.

21. Anderson LM, Brownson RC, Fullilove MT, Teutsch SM, Novick LF, Fielding J, et al. Evidence-based public health policy and practice: promises and limits. Am J Prev Med. 2005;28(5 Suppl):226-30.

22. Pappaioanou M, Malison M, Wilkins K, Otto B, Goodman RA, Churchill RE, et al. Strengthening capacity in developing countries for evidence-based public health: the data for decision-making project. Soc Sci Med. 2003;57(10):1925-37.
